# News and Notes

**Published:** 1909-02

**Authors:** 


### NEWS AND NOTES

Dr. Hugh M. Lokey, who has been associated with Dr. A. W. Calhoun since 1902, has opened offices at 412-13 Candler Building. He will confine his practice, as heretofore, to the diseases of the eye, ear, nose and throat.

Dr. E. C. Davis left recently for a trip to Panama and South America.

---

Dr. J. S. B. Holmes, formerly of Atlanta, but now of Valdosta, spent a few days with friends in Atlanta a short while since.

---

Dr. Wm. T. Bull, the noted New York surgeon, died in Savannah, February 22nd, after a prolonged illness.

---

The many friends of Dr. Yankee will be glad to learn that he is doing nicely since his recent operation of appendicitis.

---

Dr. W. C. Bryant has given up his practice in Atlanta and has entered a new field, locating at Pittsburg, Pa. During his short sojourn in Atlanta, Dr. Bryant made many friends who will regret to learn of his decision to permanently locate elsewhere.

---

Dr. John B. Deaver, of Philadelphia was a guest at a very delightful and unique dinner in Philadelphia on the night of the fifteenth. The occasion was the gathering of those members of the medical profession upon whom Dr. Deaver had done some major operation.

---

Dr. J. H. Bradfield was elected to the Atlanta Board of Health to fill the unexpired term of Mr. Erskin. (Resigned).

---

Dr. and Mrs. J. Edgar Paullin are at home to their many friends on Peachtree street, Atlanta.

---

Dr. John McArden Johnstone, one of the oldest physicians of Savannah, died February 14, 1909. Dr. Johnstone was in his eightieth year and was a veteran of two yellow fever epidemics in Savannah as well as having served in the civil war.

---

Dr. W. W. Bruce, of Kingsboro, died February 11, at the age of ninety-one years. The death of Dr. Bruce removes one of the most prominent figures of the medical profession of West Georgia.

Dr. W. B. Armstrong, one of Atlanta's most prominent physicians, died at his home on Washington street, on February 8, having been sick only a few days.

---

At a recent meeting at the Grady Hospital, the following were elected as members of the Medical staff from the Atlanta School of Medicine, from A. C. P. & S.

Medicine.—Dr. R. F. Dorsey, Dr. J. C. Olmsted, Dr. L. B. Clark and Dr. C. W. Strickler.

Surgeons.—Dr. L. C. Ficher, Dr. Goldsmith, Dr. F. K. Boland.

Obstets. and Gyn.—Dr. Geo. H. Noble and Dr. Jno. F. Ernest.

Ear, eyes, nose and throat.—Dr. R. B. Ridley, Jr., and Dr. F. C. Calhoun.

From the city at large:

Medicine.—Dr. C. G. Giddings and Dr. L. P. Stevens.

Surgery.—Dr. Walton P. Jones and Dr. J. N. Ellis.

Obstets. and Gyn.—Dr. W. A. Crowe.

Ear, eye, nose and throat.—Dr. A. W. Stirling.

---

Announcement is made in the last issue of the Chicago Clinic and Pure Water Journal that the able editor, Dr. Thomas G. Atkinson, of the Medical Standard will assume the general editorial supervision of the former journel.

The excellent work, and charming poems written by the former editor will be greatly missed by the readers of the Clinic.

---

At a regular monthly meeting of the medical board of the Grady hospital the following officers for the ensuing year were elected: Dr. Rufus T. Dorsey, president; Dr. W. S. Goldsmith, ice president, and Dr. C. W. Strickler, secretary.

A committee was appointed to draft suitable resolutions on the death of Dr. W. B. Armstrong, and read them at the next meeting of the board.

---

A Conundrum.—Into a general store of a town in Arkansas there recently came a darky complaining that a ham which he had purchased there was not good.

"The ham is all right, Zeph," insisted the storekeeper.

"No, it ain't, boss," insisted the negro. "Dat ham's shore bad."

"How can that be," continuer the storekeeper, "when it was cured only last week?"

The darky scratched his head reflectively, and finally suggested:

"Den mebbe it's had a relapse."—*Cleveland Leader.*

---

The Point.—At a dinner during the recent Episcopal convention at Richmond a young lady sitting near the bishop of London said to him, "Bishop, I wish you would set my mind at rest as to the similarity or dissimilarity between your country and ours on one point. Does the butterfly because the tomato can?" The bishop laughed heartily at this vivacious sally. Not so a young Englishman of his party, who, after dinner, sought his host. "I want to know, you know," said he, "about that joke of Miss B.'s. She asked if the butter flew because the tomatoes could. Pray tell me what the point is."—*Christian Register.*

---

### REGULAR MEETING FULTON COUNTY MEDICAL SOCIETY, NOVEMBER 19, 1908—DR. STIRLING IN CHAIR.

REPORTED BY DR. R. R. DALY.

Dr. Theodore Toepel read his paper upon "Active Exercise in the Treatment of Locomotor Ataxia," which was published in the December issue of the Journal-Record of Medicine.

It was discussed by Dr. Daly, who commended the detail and precision of the exercises described.

Dr. Thrash called attention to the distinct gain in the patient's comfort through the means described and noted the psychologic effect following the knowledge the patient gained of his capabilities.

---

### MEETING OF FULTON COUNTY MEDICAL SOCIETY, DECEMBER 3, 1908—DR. STIRLING IN CHAIR.

Dr. J. Cheston King read a paper upon "Paralysis Following Acute Disease or Due to a Specific Virus."

It was discussed by Dr. Duncan who said that after Diphtheria he had often seen regurgitation of solid as well as liquid foods. He differed from the writer as to the antecedents of tabies always being syphilitic. He knows of cases wherein no syphilis could be discovered.

Dr. Armstrong also said he knew of cases of tabes in which there was no syphilitic taint. It appeared in three generations under his observation, viz: grand-father, son and grand-daughter, and none of these had any marks or history of syphilis.

### REGULAR MEETING FULTON COUNTY MEDICAL SOCIETY, DECEMBER 17, 1908.

This was the annual meeting of the society and the President's address was read. The members received it with great satisfaction and approved emphatically many of the suggestions it contained.

At the election of officers, the following were elected: President, C. W. Strickler; vice-president, J. Ross Simpson; secretary, E. G. Ballenger; treasurer, A. H. Lindorme; Censor, M. Hoke.

Drs. Roy and Lokey were appointed to arrange for the annual banquet.

### REGULAR MEETING FULTON COUNTY MEDICAL SOCIETY, JANUARY 7, 1909, HELD AT THE ARAGON HOTEL—DR. STRICKLER IN CHAIR.

The officers elected at the previous meeting were installed and Dr. Strickler took the chair.

Dr. Stirling presented the society with a fine new gavel, which was accepted by Dr. Strickler.

Dr. Strickler then gave his inaugural address which is published elsewhere in the Journal.

Dr. Olmsted discussed the address: See special article.

Dr. Claude Smith urged that the vital statistics be better attended to by physicians. The birth rate is slowly increasing, but this may be due to better reporting. He wants the reports made so carefully that they can be depended upon.

Tuberculosis is not reported as it should be because people are not convinced that it is a communicable disease, and they fear isolation as a result of reporting. The percentage among negroes is decreasing.

Pneumonia is increasing among negroes from 32 per cent. to 08 per cent.

Typhoid fever seems more fatal recently though less in quantity.

Dr. Cartlege said in regard to quacks, that he knew of a "specialist" who was examining and treating patients free but he had made arrangements with a druggist whereby cheap drugs were sold to the patients as strange, foreign medicines at enormously high prices and the quack got his money in that way.

Dr. Hoke urged co-operation among the members in supporting a library and told of a new plan to have a special room in the Carnegie Library for the Medical books.

Dr. Niles using the classic legend of Joshua with his hand upheld by his friends, as a text, showed how the members should support the President in all his recommendations.

After adjournment the members enjoyed a banquet in the hotel dining room after which there were numerous speeches.

Credit should be given Drs. Roy and Lokey for the excellent manner in which these arrangements were made.

---

### REGULAR MEETING FULTON COUNTY MEDICAL SOCIETY HELD IN CARNEGIE LIBRARY, JANUARY 21, 1909. DR. STRICKLER IN CHAIR.

Dr. Niles read an excellent paper upon "Aesthetic Alimentation" that appears elsewhere in the Journal.

Dr. Olmsted said that none of us could fail to appreciate the prose, poetry and the splendid common sense of the article. He thoroughly agreed with the essayist upon the excellent effects of aesthetic surroundings and pleasing deliberation at meals. The happy, contented frame of mind was necessary to good digestion and good mental cultivation. He advised that the paper be published in the public prints so as to reach the people most in need of just that sort of thing. He said that the old adage, "after dinner, sit awhile," was of the soundest hygiene. The national disease of dyspepsia was due to our hurrying at eating and hastening thence to work, either physically or mentally, without giving the stomach a chance to do its work.

In closing, Dr. Olmsted paid a tribute to Dr. Niles, saying he was proud that the strenuosity of the times had not done away with the use of the literary grace in the consideration of medical topics.

Dr. Amster said he had listened with pleasure to the essay and heartily commended the idea of proper serving of food. He had noticed many times the difference in the reception patients gave food trays in the hospitals depending upon the aesthetic condition of the meals. It is a distinct advantage to have everything nice and dainty. He wanted to emphasize in addition the necessity for proper selection of food and the manner in which it should be cooked. He believes that doctors should give specific directions along this line, rather than leave the matter to the cook or family. Doctors in Europe are given instructions in cooking so as to enable them to direct their patients' dietary more precisely.

Dr. Roy said that aesthetics should be considered at the hospitals more than they were. He thought, perhaps, Dr. Amster had not noted that Dr. Niles had already taken proper selection and preparation so far as cooking went, for granted.

Dr. Niles in closing agreed with Dr. Amster as to selection and re-read a sentence in his paper showing that idea.

Dr. Stirling read his paper upon "Retro-bulbar Optic Neuritis."

Dr. Roy in discussion said that this condition is a confusing one because the apparently normal disc in the early stage seems to belie the serious condition. Also later on the white disc sometimes indicates complete blindness when the patient sees well. The classic symptoms of pain on motion of the eye, dimness of vision, central scotomata and prevented color sense ought to make the diagnosis clear and these things should be kept in mind as a warning of possibly ensuing multiple sclerosis even if the first attack were recovered from.

The prognosis of this disease is good in his estimation if treated properly during the first eight days. Later it is bad. The remedies he has found most satisfactory are iodide of potassium, mercury and pilocarpine.

Dr. Stirling in closing, said he agreed with Dr. Roy as to multiple sclerosis, but not as to the eight day limitation period of successful treatment.

Dr. Westmoreland read an exhaustive paper upon "Morbid Reflex Neurosis."

Dr. Davis said he was most impressed by neuroses depending upon deformities of the female genitalia. He told of two girls, 9 and 6 years of age, relieved of neuroses by clearing adhesions at the clitorides. Another child was always pressing her hand there because of the irritation and was about to become a masturbator when operation removed the cause.

Four of five female masturbators were cured of the habit by removal of the adhesions. He related the case of a woman who couldn't walk because of spasm in the tendon of achilles. It was relieved by the prepucial operation. As to fessure in ano, he thought that the speculum gives more force than the thumb and fingers and when judiciously used, makes the better method.

Dr. Goldsmith said that is true, there is more force in the speculum; but one loses and destroys the best guide he has when he keeps his fingers away from the muscle. He thinks Westmoreland's method gives the safe stretching to every fiber of the muscle.

He told of a case of pain in left testicle and left kidney due to organic urethral stricture that was relieved by proper treatment of stricture. He insisted that urethras should be examined with bulbaros sounds instead of the straight shank if one wanted to know anything about the size of the band. He agreed that all four operations should be done if there were the slightest indication for them: Circumcision, slitting the meatus, removing varicocele and stretching canal sphincter.

Dr. Fowler said that the pinhole meatus causes incontinence as well as retarded urination. Oxalate and uric crystals frequently were caused by meatal irritation. He told of a case of a man whose long prepuced reacted upon his nerves so that he frequently could not control feces when urinating. He recovered after circumcision.

Chronic prostatitis caused itching anus and severe mental depression in one of his cases.

Ureters were catheterized four hours in one case and caused both testicles to be drawn into their rings.

Dr. Ballenger said that diseases and irritations of the prostate gland cause a wide range of symptoms in which mental depression is prominent. He believes in thorough treatment of these surgically and then adding the psychic elements so as to encourage the patient in recovery. He gave a word of warning as to over-zealous meatotomy. He knows of patient cut so widely open as to have to sit down when he urinates in order to keep the stream in the proper vessel.

Sexual neurasthenics are to be helped by relief in these parts and the proper advice following.

Dr. Olmsted said that he recalled the days when there was the beginning of much cutting of the meatus and several were too wide. The Otis sound is the only safe guide as to the condition in his estimation and the incisions should be deep enough to remove the ring when it extended beyond the mucous surface. Spasmodic strictures have been operated upon through the perinaeum before the proper examining methods were evolved. He told of a case of a small boy seemingly paralyzed and wearing some sort of apparatus upon his leg, who was cured of all trouble by circumcision and meatotomy.

Dr. Lokey told of boys suffering with dimness of vision, but having no refractive trouble, cured by circumscision.

Dr. Visanska told of some gastro-enteric difficulties in children cured by circumcision when direct remedies had failed.

Dr. Westmoreland said in closing that if membrane at meatus is thick it must be cut as deeply as it goes and the thicker it is the more must be allowed for subsequent cicatricial contraction. From 1 to 3 millimeters are needed. Moreover, he always re-examines after recovery and if there isn't enough room, he cuts again. He told of a case of renal calculus caught in meatus. Meatus was enlarged and calculi did not recur. This may have been due to freer urination and no retention of amorphous urates.

Withdrawing before sexual connection is complete and the use of condrums cause congestion of parts for which there is no cure except cessation of the habit.

Adhesions at prepuce should be broken up because they sometimes cause carcinonia.

Aphonia and spasm of oesophagus have been relieved by removal of prepuce.

